# Internal Inflammation of the Eye of the Horse

**Published:** 1896-09

**Authors:** 

**Affiliations:** Chief veterinarian in Arad


INTERNAL INFLAMMATION OF THE EYE OF THE HORSE. (By Kirnbauer, chief veterinarian in Arad.) The frequent tendency of horses in Lower Hungary, and especially in the province of Banat, to a general internal inflammation of the eye followed by blindness, has led me to pay more strict attention to this disease. It is a fact not to be contradicted that the disease appears much oftener here than elsewhere, and that its intensity in regard to destruction of the sight is much greater here than in other provinces. While it often happens in Siebenbürgen that the supply of cavalry horses from Szegedin after overcoming general inflammation of the eye, only on repeated attacks, after one to two years, suffer such clouding of the lens that sight is destroyed and the disease is confined to one eye, in Lower Hungary the attacks of inflammation are of such intensity that clouding of the lens of the eye is plainly visible after the first attack and loss of sight follows often in one or two months. As a rule, both eyes are either attacked at the same time or it spreads in a short time from one to the other, so that total blindness soon results. In regard to the cause nothing positive can be adduced, the inherited disposition to blindness seems to play the principal rôle; possibly the climate may have something to do with it; one of the principal factors may be the dusty condition of the roads and fields in summer, often lasting for months. The eyes would at least be weakened by this and affected mechanically, and perhaps also chemically, so that there would be, as a consequence, a previous predisposition to inflammation.

From January 1st until the end of June, 1895, twenty-six horses in all in the army service were mustered out, mostly on account of cataract on both eyes, and partly on account of periodical inflammation of one or both eyes. Since nothing certain has as yet been ascertained in regard to the cause of this disease, which destroys the utility of a horse for cavalry purposes, it seems absolutely necessary that on examination of the horses the eyes should be very rigorously examined with the aid of the ophthalmoscope, and every horse which shows the least defect of the eyes or evidence of once having had ophthalmic inflammation be rejected.

I will now cite some cases which I have observed: On December 5, 1894, I examined with the ophthalmoscope the eyes of the horses in a cavalry supply and found in a cavalry horse, "Nézö," contracted pupils, a light apple-green tapetum lucidum, a little stronger distention of the chambers around the papilla nervi optici, and the eyelids somewhat puffed up. The horse was attacked in January, 1895, with ophthalmitis universalis of the right eye, and had in March, 1895, a pronounced gray cataract.

In a second cavalry horse, "Nadbot," contracted pupils, especially the right, the tapetum lucidum in patches, light colored, the pupils reacted well to atropine. This mare was attacked in March, 1895, and eight weeks afterward went blind of cataract.

In a third horse, "Ocksa," which I examined three days after it had arrived, I found the pupils contracted and reacting very slowly on alternating light and darkness. No reaction was visible for two hours after an application of atropine, and I made a second, which was without result. The next morning I applied a fresh solution, and to my astonishment both pupils were as round as a circle, and the iris formed strips around them of about 3 mm. in width. Internally I could detect no signs of disease with the ophthalmoscope. Since the eyes, however, on account of their sunken position and contracted pupils seemed suspicious, I made two more examinations with the ophthalmoscope, but found nothing to confirm my suspicions. On the thirty-seventh day the horse had a light attack of periodic inflammation in both eyes and is at present verging upon blindness.

On the other hand, certain cases occur where, in spite of certain changes in the interior of the eye, blindness does not follow for a long time. For example, a horse, which I examined for the first time in 1890, had a well-defined triangular-shaped clouding in the middle of the lens capsule of the left eye, a little further toward the external edge of the pupil; each side of the triangle was about 45 mm. long. Three years later I saw the horse again. The clouding was unchanged in the same spot and the eye otherwise healthy. Three months later the horse died of anthrax.

A second case came to my notice in March, 1895; the horse had in the left eye a long adhesion thin as a hair, and a second somewhat thicker crossing it in the shape of an inverted T. During the eleven months the horse has been here it has not been troubled with any disease of the eye.

The last two cases show that in making a prognosis a certain reserve is always to be observed. On the other hand, the other cases make it clear that a professional man should never allow the purchase of a horse whose eyes present the slightest internal inflammation.

In conclusion I will add that I have become acquainted with a great part of the horses of southern Hungary through examining them, and that from 8 to 10 per cent. of the horses which are otherwise acceptable must, after being examined with the ophthalmoscope, be rejected on account of some defect in the eyes. Since there is hardly any disease which incapacitates a larger number of good and young horses than this periodic inflammation, it is to be recommended that military veterinarians endeavor to have a course on the use of the ophthalmoscope, similar to the one on bacteria, introduced into the veterinary schools at Vienna, Lemberg, and Buda-Pesth, and that only such veterinarians as are proficient enough be appointed on the examining boards.—*Thierärzt. Centralblatt*, May 1, 1896.

